# Exploring open science practices in behavioural public policy research

**DOI:** 10.1098/rsos.231486

**Published:** 2024-02-21

**Authors:** Maximilian Maier, František Bartoš, Nichola Raihani, David R. Shanks, T. D. Stanley, Eric-Jan Wagenmakers, Adam J. L. Harris

**Affiliations:** ^1^ Department of Experimental Psychology, University College London, London, UK; ^2^ Department of Psychological Methods, University of Amsterdam, Amsterdam, The Netherlands; ^3^ Deakin Laboratory for the Meta-Analysis of Research (DeLMAR), Deakin University, Burwood, Australia; ^4^ Department of Economics, Deakin University, Burwood, Australia

**Keywords:** nudging, open science, pre-registration, public policy, selective reporting

## Abstract

In their book ‘Nudge: Improving Decisions About Health, Wealth and Happiness’, Thaler & Sunstein (2009) argue that choice architectures are promising public policy interventions. This research programme motivated the creation of ‘nudge units’, government agencies which aim to apply insights from behavioural science to improve public policy. We closely examine a meta-analysis of the evidence gathered by two of the largest and most influential nudge units (DellaVigna & Linos (2022 *Econometrica* **90**, 81–116 (doi:10.3982/ECTA18709))) and use statistical techniques to detect reporting biases. Our analysis shows evidence suggestive of selective reporting. We additionally evaluate the public pre-analysis plans from one of the two nudge units (Office of Evaluation Sciences). We identify several instances of excellent practice; however, we also find that the analysis plans and reporting often lack sufficient detail to evaluate (unintentional) reporting biases. We highlight several improvements that would enhance the effectiveness of the pre-analysis plans and reports as a means to combat reporting biases. Our findings and suggestions can further improve the evidence base for policy decisions.

## 1. Introduction

Nudging is one of the most widespread applications of behavioural science to public policy. Nudge theory postulates that small changes in choice architecture substantially influence real-world decision-making [1]. Unlike most other forms of influence, nudges maintain freedom of choice by not restricting choice options. The popularity of nudges has motivated the creation of nudge units: government agencies or independent companies that evaluate different behavioural interventions to inform decisions on whether to roll them out more widely (more than 200 nudge units in more than 40 countries have been created to date ([2], fig. A1)). Nudge units aim to deliver substantial policy benefits with comparatively small interventions [3].

The UK Behavioral Insights Team (BIT), founded in 2010 and the oldest and largest behavioural insights team, has completed more than 1000 projects.^1^ The BIT website lists 137 reports and 36 publications, usually produced in collaboration with government agencies. There are a number of success stories among these projects, where considerable real-world benefits have been delivered. In one trial, for example, BIT used behavioural insights to design better tax reminder messages using social norms, leading to increased average payments [4].^2^ BIT is a large multi-national organization, with offices in multiple countries, including the UK, Canada, the USA, France, Australia and Singapore. It was formed within the UK government but is now a social purpose organization operating outside the government. In the USA, the Office of Evaluation Sciences (OES) was established by a Presidential Executive Order in 2015 with the mission to rigorously test and incorporate behavioural insights into government agencies. OES has completed over 90 impact evaluations affecting the lives of millions of citizens.^3^ Compared with BIT, OES is a comparatively small team that operates within the US government. Crucially, behavioural science units use randomized controlled trials (RCTs)—the ‘gold standard of evaluation’. For example, BIT has completed more than 700 RCTs to date in many different countries.^4^ This adoption of RCTs has enhanced the evidence base for government policy.^5^

The nudge approach is not, however, without critics [6]. Two main objections are: (i) despite the aforementioned success stories, overall evidence for the effectiveness of nudges in the academic literature is weak [7–9]; and (ii) nudge-based interventions may detract from more systemic reforms [6]. These criticisms culminated in a recent manifesto for applying behavioural science [10], proposing a variety of reforms and calling for ‘increased self-scrutiny’. Following these calls, we take a close look at the distribution of test statistics and safeguards against biased reporting in nudge unit trials. We argue that nudge units can further enhance their current practices with specific improvements in the transparency of their trial registration, reporting and data sharing.

## 2. Exploring potential reporting biases in nudge unit trials using bias correction techniques

DellaVigna & Linos [2] collected a large dataset of nudge unit interventions run by OES and BIT North America (126 randomized control trials covering 23 million individuals)^6^ and compared them with trials in academic journals to evaluate the shrinkage of effects when applied at scale. The comparison showed that the average impact of nudges reported in academic journals (8.7 percentage points increased take-up, a 33.4% increase over the average in the control condition) was larger than in trials run by OES and BIT (1.4 percentage points increased take-up, an 8.0% increase over the control condition). This was primarily attributed to selective publication and low statistical power in the academic studies. Although with smaller effect sizes, the nudge unit interventions were found to produce reliable, ‘sizable and highly statistically significant’ [2, p. 81] effects. Importantly, DellaVigna & Linos [2] assumed no selective reporting in the nudge unit interventions because they obtained access to the comprehensive record of trials. In addition, they visually inspected the distribution of *t*-statistics and conducted a regression test for funnel plot asymmetry, testing both the relationship between minimum detectable effect and treatment effect, as well as between standard error and treatment effect. Both the visual inspection of *t*-statistics and the regression indicated no evidence for publication bias.^7^

However, while the comprehensive record of trials protects from publication bias (when a complete study is omitted), it does not necessarily protect from other forms of selective reporting (e.g. choosing which outcome variables to report or emphasize, or what covariates to include). Further, both visual inspection of funnel plots, as well as regression of effect sizes on standard errors, have been shown in simulation studies and empirical examples to often have low power to detect reporting biases especially under high heterogeneity [11,12]. Here, we therefore apply statistical techniques that are more suitable to test for potential reporting biases in the presence of heterogeneity to the nudge unit dataset [13] (i.e. 241 nudges from 126 trials, as collected in [2]).

DellaVigna & Linos [2] extend the standard meta-analytic framework by modelling the effect sizes as a two-component random effects meta-analytic mixture. This means that instead of assuming that all effects come from a single distribution, as is common in meta-analyses, their framework allows the effect sizes to come from two separate distributions. Prima facie, such an approach seems reasonable given the large differences between different behavioural interventions incorporated under the term ‘nudge’ [8]. For example, researchers might assume that effect sizes for nudges that change the default option are distributed differently from nudges with smaller effects.^8^ Here, we follow DellaVigna & Linos and take a data-driven approach to determine the appropriate number of distributions. Models assuming a single distribution (i.e. the standard meta-analytic random effects model) are compared with models with larger numbers of mixture components using model selection techniques to find the appropriate model. DellaVigna & Linos [2] show that assuming all effect sizes come from a single distribution does not adequately describe the data. We come to the same conclusion when comparing single-component models and mixture models using Bayesian information criterion (BIC). For this reason, and to keep our analysis comparable to that of DellaVigna and Linos, we proceed with the mixture modelling approach.

To assess selective reporting via bias correction methods, we extended DellaVigna & Linos’ [2] analysis in three ways. First, we allow for moderation by domain within the mixture model (i.e. different areas in which nudges may be used, such as work and education or healthcare, as classified in [2]). This is important, as inclusion of appropriate study-level covariates may explain some of the non-normal heterogeneity that would otherwise be captured by using multiple mixture components.

Second, we additionally specify mixture models that allow for selective reporting—selection models—and compare them with the normal models. Selection models, as we specify them here, include an assumption that null or backfire effects are suppressed within the distribution of effects reported. We include three types of selection models: (i) models that assume that negative results are less likely to be published than positive results, (ii) models that assume that non-significant studies at *α* = 0.10 are less likely to be published than significant studies, and (iii) models that assume that non-significant studies at *α* = 0.05 are less likely to be published than significant studies. We used BIC-based Bayesian model averaging to combine the evidence across the three types of selection models [14,15].

Third, we also allow expansion to three-component mixtures. This may improve model fit further compared with the two-component results.^9^

Overall, we fit the following six models to the full dataset (more details on the specified models are provided in the electronic supplementary material):

(i) a random effects meta-analytic model (normal model);
(ii) a two-component random effects meta-analytic mixture model (2-mixture), as in DellaVigna and Linos [2];
(iii) a three-component random effects meta-analytic mixture model (3-mixture);
(iv) a random effects meta-analytic model with adjustment for selective reporting (selection model);
(v) a two-component random effects meta-analytic mixture model with adjustment for selective reporting (selection 2-mixture), following DellaVigna and Linos [2];
(vi) a three-component random effects meta-analytic mixture model with adjustment for selective reporting (selection 3-mixture).

We estimate the models using the optim() optimization routine from the optim package in R ([17], v. 4.3.2; Windows 11).

**Figure 1.**
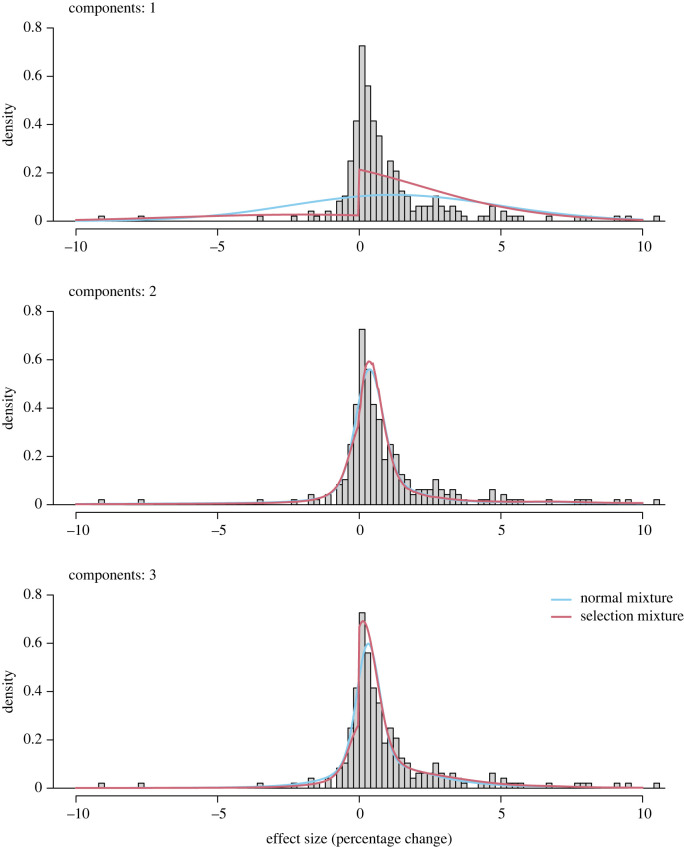
Distribution of all effect sizes and visualization of the meta-analytic models. One effect size smaller than −10 and six effect sizes larger 10 are not shown.

Figure 1 visualizes the model-fit of the different models to the full dataset. When looking only at mixtures of one and two components (matched to DellaVigna & Linos [2]), we find that the data are most in line with a model assuming a mixture of two normals and selective reporting (BIC weights: normal model, 0.000; normal 2-mixture, 0.042; selection model, 0.000; selection 2-mixture, 0.958). The figure also clearly indicates that a single normal distribution does not capture the data well, which suggests that different types of nudges are described by different distributions.^10^ When we also allow extension to three parameter mixtures, we find somewhat weaker evidence for selective reporting; however, most weight is still given to the selection 3-mixture, a model that assumes selective reporting (BIC weights: normal model, 0.000; normal 2-mixture, 0.000; normal 3-mixture, 0.249; selection model, 0.000; selection 2-mixture, 0.000; selection 3-mixture, 0.750).

We can also make inferences about the type of publication bias based on which types of selection models received the highest weight. This shows that the lowest BIC was given to the three-component selection model, which assumes that results with negative estimates (rather than non-significant results) are suppressed. This model has the lowest BIC when looking at one-component models, and the second lowest when looking at two-component models (with the *α* = 0.10 model being slightly preferred). Overall, this suggests that selective reporting operates most strongly on suppressing backfire effects rather than on selection for *p* < 0.05.^11^ We next directly compared pre-analysis plans with publicly available final reports. This enables us to identify instances where pre-analysis plans may allow for selective reporting and provide corresponding recommendations.

## 3. Pre-analysis plans leave scope for selective reporting

Both the BIT and OES document their intended analyses in pre-analysis plans. This is laudable, diminishes the scope for selective reporting and enables evaluation of any deviations from such plans (if they are shared publicly). However, previous research comparing trial protocols or pre-registrations with corresponding published journal articles indicates that selective *reporting* is still possible, even without selective *publication* (e.g. in economics: [18]; in medicine: [19]; in psychology: [20]). Selective reporting practices include choosing which outcome variables to report or emphasize and what covariates to include. These practices can be (and probably usually are) unintentional—it is easy for any researcher to convince themselves that the analysis with covariate A is ‘most appropriate’ once knowing the outcome, without recognizing the potential for bias in such a decision [21].

Below, we investigate whether the pre-analysis plans of trials run by nudge units allow for selective reporting. We (i) evaluate how detailed the pre-analysis plans are and whether they cover all relevant researcher degrees of freedom, and (ii) compare pre-analysis plans with published reports to assess selective reporting. While we were unable to obtain the pre-analysis plans from BIT (in the UK or US), despite taking a variety of steps,^12^ OES trial protocols are publicly available. We searched for the 50 most recent pre-analysis plans, as of August 2022, and compared them with the final published reports. We excluded reports that did not include pre-analysis plans or did not include results (for example, because OES could not obtain the necessary data). We further skipped two trials that had conflicting registrations on OES and ClinicalTrials.gov, leaving us with a final sample of 32 reports with corresponding pre-analysis plans (see electronic supplementary material for details).

The open access publication of all OES pre-analysis plans is exemplary and represents best practice. Such transparency enables appropriate evaluation of the reliability of the data obtained. Before we proceed with our evaluation, it is important to acknowledge that the enabling of such an evaluation is in itself a positive outcome of such practices.

**Figure 2.**
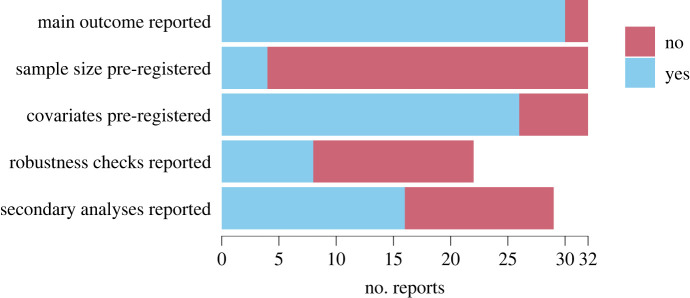
Aspects of pre-analysis plans of trials run by nudge units.

Figure 2 summarizes the results of our evaluation. Our evaluation demonstrates several additional examples of best practices in OES pre-analysis plans. Some plans are highly detailed, including analysis scripts for later analyses (e.g. https://web.archive.org/web/20240110143223/; https://oes.gsa.gov/projects/soar/). Additionally, 30/32 final reports at least detail the main outcome as described in the analysis plan, or otherwise disclose it transparently. There is, however, large variability in the quality of the pre-analysis plans and several plans have limitations. In particular, we find that both pre-analysis plans and final reports are usually insufficiently detailed to determine whether any selective reporting has taken place. 28/32 pre-analysis plans lack a sample size specification. This allows for ‘optional stopping’, where data is collected until a statistically significant result is found—a practice likely to inflate type 1 error rates [21,22]. While OES often uses existing data, and therefore sample size justification may not be applicable, we noted that often the nature of the existing data (e.g. which agency will supply it or in which time period it will be collected) was not clear. Further, the data are generally not publicly available. In many cases, this will be for sound legal reasons. However, making anonymized data available wherever possible is good practice, as it allows other researchers to independently verify the claimed results and conduct additional robustness checks [23].

In 6/32 analysis plans, information about the covariate inclusion was lacking. This allows for analytic flexibility, where covariate specifications can be explored until a statistically significant result is found—again a practice that may inflate type 1 error rates [21,22]. For example, one analysis plan^13^ determines demographic covariates to include in the regression for the main outcome as follows: ‘The precise way these demographic variables are categorized and included in the specification is not defined here.’ While this plan also includes a robustness check without covariate adjustment, the results of this check are not reported, and it is not possible to know which covariates are included for the test included in the final report. Indeed, 14/22 reports that pre-register robustness checks do not contain the outcomes of those checks, and it is generally difficult to identify which covariates were included when estimating reported effect sizes. Finally, in 13/29 cases whose pre-analysis plans specify secondary analyses, these are not included in the final reports.^14^

Overall, those pre-analysis plans that have been shared are insufficient to rule out optional stopping or (intentional or unintentional) *p*-hacking. However, it is important to emphasize that this does not imply that, therefore, optional stopping or *p*-hacking has taken place—only that the existence of analysis plans does not strictly rule them out.

## 4. Evidence-based public policy needs to increase transparency

We find that the pre-analysis plans and final reports lack sufficient detail to evaluate whether selective reporting has occurred, while statistical techniques provide suggestive (but not conclusive) evidence for reporting biases. We call for more transparency, so that the quality of the work by nudge units can be independently evaluated by other researchers. Similar to recommendations for pre-analysis plans and transparency in academia (where similar problems have been identified; [18–20,24]), nudge units may increase transparency by taking several steps (roughly ordered by ease of adoption):

(i) Analysis plans should be shared publicly by all nudge units.^15^ OES should be applauded for already doing so.
(ii) Analysis plans should be specific and include covariates for regression specification and either planned sample size or detailed information about the existing dataset being used. In our supplements, we provide a recommendation for an updated OES analysis plan template that includes a specific section for sample size and treatment of covariates. In some cases, the dataset may not yet be shared with the nudge unit itself when the analysis plan is created. In these cases, it may not be possible to specify covariates in advance, or the anticipated covariates may be different from the ones available later. It is then important to be transparent about which covariates are included in the model and how they deviate from the analysis plan. Further, sensitivity analyses will then help to understand robustness to the covariate structure.
(iii) Write-ups of trial outcomes should be shared publicly and report the outcomes of all statistical tests that were specified in the pre-analysis plans. In general, more detail about the conducted analyses needs to be provided than is currently the case. If the main write-ups are intended to be short and for non-experts, another document with all analyses that were conducted should be shared (e.g. an R Markdown file).
(iv) The anonymized data and analysis code should be shared publicly. We are aware that this may not be possible in many cases (e.g. when medical records are used); however, currently virtually no data are shared. We therefore urge BIT and OES to make the anonymized data available where this is legally possible.
(v) Independent audits by third parties should take place to compare the pre-analysis plans against the reports. For example, behavioural insights teams could give small monetary awards to anyone who detects a mismatch between a published pre-analysis plan and corresponding report (similar to red team approaches that have been successfully applied in academia).^16^ Further, government agencies and other contractors should include an evaluation of the work, when commissioning BIT or OES to run a trial (e.g. the UK Cabinet Office could fund PhD students to compare write-ups and pre-analysis plans). Note that it should be considered completely appropriate to deviate from an analysis plan or conduct additional analyses so long as deviations are transparent and justified and if confirmatory and exploratory analyses are clearly delineated in the report [25].

One potential response to our suggestions is that BIT is a private company and thus should not be required by law to share pre-analysis plans or reports. While this may be a valid view, we point out that in most cases, BIT is in fact contracted by Government agencies. In these cases, where the taxpayer funds the research conducted, the contract should require the sharing of pre-analysis plans and of the outcomes of the research.

Further, we want to emphasize that nudge units have made an important contribution by popularizing RCTs within government. This allows researchers and policymakers to evaluate the effectiveness of different policy interventions and is an important pillar of evidence-based policy-making. We do not see our criticisms as showing the limitations of RCTs in general but only aim to point out specific and feasible improvements that nudge units could make to further enhance the effectiveness of their valuable work.

There is great benefit in applying evidence-based behavioural science to public policy evaluated with randomized controlled trials, and there are many examples of evidence from behavioural science positively affecting policy [26]. We also point out that OES has already taken several steps to increase transparency that go beyond many other government agencies. The inclusion of publicly accessible pre-analysis plans by all nudge units is a further step towards gold standards in behavioural science application. The evaluation we present is intended to motivate further strides towards fully transparent, evaluable, high-quality research. We are confident that applied nudge units can embrace this challenge to the further benefit of society.

## Data Availability

All data and materials are available at https://osf.io/f3rxt/ [27]. Additional results are provided in electronic supplementary material [28].

## Footnotes

1 https://web.archive.org/web/20240108153747/; https://www.bi.team/about-us-2/who-we-are/.

2 However, this effect failed to replicate in a different council [5].

3 https://web.archive.org/web/20240117111129/; https://oes.gsa.gov/work/.

4 https://web.archive.org/web/20240108153747/; https://www.bi.team/about-us-2/who-we-are/.

5 For example the European Commission states about behavioural insights: ‘In practice, however, behavioural insights mainly contribute to the impact assessment process. This process consists in gathering and analysing evidence about the likely impacts of a planned policy.’ See https://web.archive.org/web/20240110142327/; https://knowledge4policy.ec.europa.eu/behavioural-insights/about-behavioural-insights_en.

6 This is less than the number quoted in the introduction as many BIT trials have been conducted outside the USA.

7 By contrast, in online appendix A2, DellaVigna & Linos [2] show that published articles based on the nudge unit interventions suffer from the same pattern of publication bias as published academic papers.

8 The difference between these types of interventions could also be modelled by including appropriate moderators. However, often researchers do not know all the relevant differences between nudge characteristics *a priori.* This is also the case in [2], which found evidence for mixtures despite including different moderators in the analysis.

9 Two reviewers suggested applying the robust Bayesian meta-analysis (RoBMA) method [11,16]. RoBMA like most other ‘out-of-the-box’ meta-analytic methods, assumes that effect sizes follow a single distribution. Extending RoBMA, and other meta-analytic methods, to mixture modelling is a non-trivial endeavour (computational tractability, convergence, parameterization etc.), and therefore we proceeded with analysis analogous to DellaVigna & Linos [2].

10 In line with DellaVigna and Linos, we use the mixture model here to obtain a better non-parametric approximation of the distribution shape. It would be an interesting project to develop further the theoretical interpretation of each of the components, but this lies beyond the scope of the present article.

11 An important consideration for future research is how the mixture models and the publication bias models may interact. Including the mixture model does weaken the evidence for selective reporting (as visible in the BIC differences in electronic supplementary material, appendix 3) and it may be that the mixtures can approximate patterns of selective reporting to some extent.

12 First, we contacted the head of US BIT to ask for the protocols. Second, we tried to obtain the protocols via DellaVigna and Linos, who recommended contacting BIT directly. Third, we contacted the UK BIT through a form on their website and received an initial response, but this did not follow through to sharing the pre-analysis plans. Fourth, we tried to obtain the protocols through a Freedom of Information request at https://web.archive.org/web/20240117111819/; https://www.whatdotheyknow.com/request/trial_protocols_behavioural_insi/#incoming-2143267 and https://www.whatdotheyknow.com/request/trial_protocols_behavioural_insi#incoming-2143267 and after this request was rejected because the workload would be too high, we created another request targeting a shorter timespan https://web.archive.org/web/20240117112028/; https://www.whatdotheyknow.com/request/trial_protocols_behavioural_insi_2 and https://www.whatdotheyknow.com/request/trial_protocols_behavioural_insi_2. This was also rejected with the justification that determining whether the department holds the information, locating, retrieving and extracting it would take more than 3.5 days.

13 https://oes.gsa.gov/projects/transparent-defaults/.

14 We also contacted the Office for Evaluation Sciences as well as DellaVigna & Linos to ask whether more detailed reports are available but received no response.

15 We are aware that sometimes contracts with clients may not allow doing so; however, we believe this is unlikely to be the case for all trials run (BIT), and recommend contracting in a way that allows sharing the pre-analysis plan, which is ultimately also in the interest of the clients.

16 https://web.archive.org/web/20240110143330/; http://daniellakens.blogspot.com/2020/07/the-red-team-challenge-part-3-is-it.html.
